# Effects of flanker size and flanker eccentricity on the spatial integration of orthographic information

**DOI:** 10.3758/s13414-026-03293-w

**Published:** 2026-06-11

**Authors:** Christophe Cauchi, Jonathan Mirault, Bernard Lété, Jonathan Grainger

**Affiliations:** 1 Analyse Et Traitement Informatisé de La Langue Française (ATILF), Université de Lorraine & CNRS, Office J218, Campus Lettres Et Sciences Humaines, 23, Boulevard Albert 1er, 54 000 Nancy, France; 2 Centre de Recherche en Psychologie Et Neurosciences, Aix-Marseille Université and CNRS, Aix-en-Provence, France; 3 Laboratoire d’Études Des Mécanismes Cognitifs, Université Lumière Lyon 2, Lyon, France; 4 Pôle Pilote AMPIRIC, Institut National Supérieur du Professorat Et de L’Éducation (INSPE), Aix-Marseille Université, Aix-en-Provence, France; 5 Institute for Language, Communication, and the Brain, Aix-Marseille Université, Aix-en-Provence, France

**Keywords:** Flanking letter effects, Lexical decision, Flanker eccentricity, Flanker size

## Abstract

We investigated the impact of flanker size and horizontal flanker eccentricity in the reading version of the flankers task. Critical targets and the corresponding flankers were words that could either be the same or different. Target size did not change, and flankers could either be the same size as targets or larger. Flankers could be located close to targets (normal spacing – close flankers) or separated from targets by seven spaces (distant flankers). Results revealed significant effects of flanker relatedness (same word as the target or a different word) that interacted with both flanker size and flanker eccentricity in the analysis of response times. The three-way interaction was not significant. The only significant effect in the analysis of error rates was that of flanker relatedness. We replicated prior findings showing that increasing flanker eccentricity diminishes the effect of flanker relatedness, and we further demonstrated that increasing flanker size increases the magnitude of flanker effects. Crucially, an increase in flanker size was not found to compensate for the negative impact of an increase in flanker eccentricity on effects of flanker relatedness. These results suggest that eccentricity is one key visual factor influencing the spatial integration of orthographic information, and that the impact of eccentricity overrides the impact of flanker size. This provides support for the hypothesized scale-invariant gaze-centered processing of letter identities during the initial phase of orthographic processing.

**Supplementary Information:**

The online version contains supplementary material available at 10.3758/s13414-026-03293-w.

## Introduction

There is growing evidence that information, and in particular orthographic information, spanning multiple words is processed in parallel during sentence reading (see Snell & Grainger, 2019, for a review). Furthermore, there is evidence that orthographic information extracted from parafoveal words influences processing of the fixated foveal word (we refer to this as the spatial integration of orthographic information; see Kennedy & Pynte, 2005, for an earlier demonstration of a different type of parafoveal-on-foveal effect). One key question, therefore, is precisely how this spatial integration of orthographic information operates and what factors influence this process? In the present study we focused on how purely visual factors – specifically character eccentricity and size – modulate the spatial integration of orthographic information during the processing of multiple words. To do so, we used the reading version of the flankers task introduced by Dare and Shillcock (2013). In Dare and Shillcock’s Experiment 1, participants performed a lexical decision task on centrally located words and nonwords. The novelty in that the study involved the presence of flanking letters located either side of targets, and either related or not to targets (e.g., RO ROCK CK vs. PA ROCK TH). Dare and Shillcock found that related flankers facilitated lexical decisions to central targets (see Grainger et al., 2014, for a replication and extension of the results of this seminal study, and an explanation of the results).

Since the work of Dare and Shillcock (2013) and Grainger et al. (2014), a number of studies have attempted to shed light on the mechanisms driving such flanker effects and what factors might modulate these effects (e.g., Cauchi et al., 2020; Lázaro et al., 2025; Lázaro et al., 2026; Snell & Grainger, 2018; Snell & Simon, 2025). For example, Snell and Grainger (2018) manipulated the position and relatedness of word flankers (left or right of targets, or bilaterally) and found a distinct rightward bias in the effects of flanker relatedness. They attributed this to the well-established rightward attentional bias when reading languages that are read from left-to-right (see Rayner, 1998). However, more recent work by Lazaro et al. (2026) has revealed a leftward bias when flankers are single letters, thus pointing to a key role for initial letters in orthographic processing (see also Grainger et al., 2016).

Fournet et al. (2023) manipulated the number of nonword flankers – either two bigram flankers (e.g., CK RO ROCK CK RO) or quadrigram flankers (e.g., CKRO ROCK CKRO), which again were either related or unrelated to the central target. One key finding in that study was that only proximal flankers had an impact on target processing, and that adding spatially distinct flankers was detrimental to effects of flanker relatedness, possibly by an increased spread in spatial attention. The term “proximal flankers” refers to flankers that were immediately adjacent to the target word, whereas distal flankers were separated from the target by intervening flanking stimuli.

However, with respect to the present study, the key finding of Fournet et al. (2023) was from their Experiment 2, where flanker eccentricity was manipulated (either one or two character spaces from the edge of targets: e.g., RO ROCK CK vs. RO ROCK CK). Effects of flanker relatedness were found to be significantly greater for the close flankers. Following up on this finding, Cauchi and Meeter (2025) used a more extreme manipulation of flanker eccentricity (nine levels of eccentricity) with word flankers that could be related or not to the central target word. An eye tracker was used to ensure that flanker stimuli were displayed during participants’ central fixation on the target stimulus. The results demonstrated that there were significant flanker effects with flankers located up to 4.3 degrees of visual angle from targets, which corresponded to a distance of 15 character spaces. This demonstrated that orthographic information can be extracted from peripherally located flanker stimuli (when these are words) at rather large distances from fixation, and when there are no stimuli intervening between targets and related flankers.

Another important follow-up to the work of Fournet et al. (2023) was provided by Mlinarič et al. (2025), who examined the impact of multiple flankers (three flankers on each side of targets), but this time with word flankers (flankers were four-letter words and critical targets were four-letter words). Related flankers (i.e., the same word as targets) could occur at one of the six flanker positions. This manipulation allowed the authors to test whether orthographic information from non-adjacent (distal) word flankers could influence target processing. Their results showed reliable facilitation effects only for flankers immediately adjacent to the target (i.e., proximal flankers), with no measurable influence of distal flankers. Therefore, in line with the findings of Fournet et al., Mlinarič et al. found that only proximal flankers impacted on central target processing. The findings of Fournet et al. (2023) and Mlinarič et al. (2025), combined with the results of studies manipulating flanker eccentricity with single flankers (Cauchi & Meeter, 2025; Fournet et al., 2023), suggest that the processing of more eccentric flankers is disrupted by the presence of other flanking stimuli intervening between the eccentric flanker and targets. Fournet et al. (2023) concluded that the addition of intervening unrelated flankers between related flankers and targets caused an increase in the spread of spatial attention hence reducing effects of flanker relatedness.

A related line of research in vision science has highlighted the central role of perceptual constraints on letter recognition in shaping how orthographic information is extracted across the visual field. For example, Legge et al. (2001) showed that the number of letters that can be reliably identified in a single fixation decreases markedly with increasing retinal eccentricity, and that this reduction of the visual span directly limits reading performance in peripheral vision. Importantly, their work also demonstrated that letter size modulates reading speed at all eccentricities, with larger print partially compensating for peripheral limitations up to a critical print size (see Grainger et al., 2016, for a discussion of the role of visual factors in orthographic processing).

Complementary evidence for the role of visual factors in orthographic processing comes from studies of visual word recognition showing that word processing varies systematically as a function of fixation position within a word, a phenomenon known as the viewing position effect (VPE; Nazir et al., 1998) – that is, word recognition is facilitated by initial fixations closer to the center of the word (often referred to as the “optimal viewing position effect” or OVP; e.g., O’Regan & Jacobs, 1992). Moreover, the magnitude of the VPE was shown to be closely linked to letter legibility (see also Stevens & Grainger, 2003), and that magnifying letter size as a function of eccentricity can attenuate the VPE for short words, although strong effects of letter eccentricity persist for longer words even when attempting to homogenize letter legibility by increasing the size of the most eccentric letters. This research therefore reveals the limits in the extent to which increased physical salience (by increasing letter size) can compensate for the detrimental effects of eccentricity (although it remains to be seen if manipulations of physical salience other than letter size might be more effective).

Above, we noted that research using the reading version of the flankers task has shown that increasing flanker eccentricity reduces their influence on central target word recognition (hence mimicking the effect of letter eccentricity on word recognition, as discussed in the preceding paragraph). However, it remains unclear whether the physical salience of parafoveal stimuli – manipulated through their size – could compensate for the limitations imposed by eccentricity. In other words, the question addressed in the present study is: Can increasing the size of distant flankers increase their ability to impact on target processing? Addressing this question is critical for understanding how perceptual constraints (e.g., eccentricity, size) influence effects of flanker relatedness in the reading version of the flankers task.

Thus, with the aim of addressing this question, in the present study we added a manipulation of flanker size to the manipulation of flanker eccentricity. Moreover, we used word flankers, as in the study of Mlinarič et al. (2025), as opposed to the bigram flankers tested by Fournet et al. (2023). Therefore, our experimental design involved three two-level factors: Flanker Size (same as target vs. larger than target), Flanker Eccentricity (close vs. distant), and Flanker Relatedness (related vs. unrelated). Table 1 summarizes the conditions tested in the present study. Target size was held constant across all conditions. We predicted that both flanker size and flanker eccentricity should impact on effects of flanker relatedness. Crucially, we were interested in knowing whether an increase in flanker size could compensate for an increase in flanker eccentricity as a test of the impact of purely visual factors in driving flanker effects. In other words, we examined whether increasing the size of parafoveal stimuli could compensate for the reduction in flanker effects typically observed when flankers are presented at greater eccentricities (Cauchi & Meeter, 2025; Fournet et al., 2023).

**Table 1:**
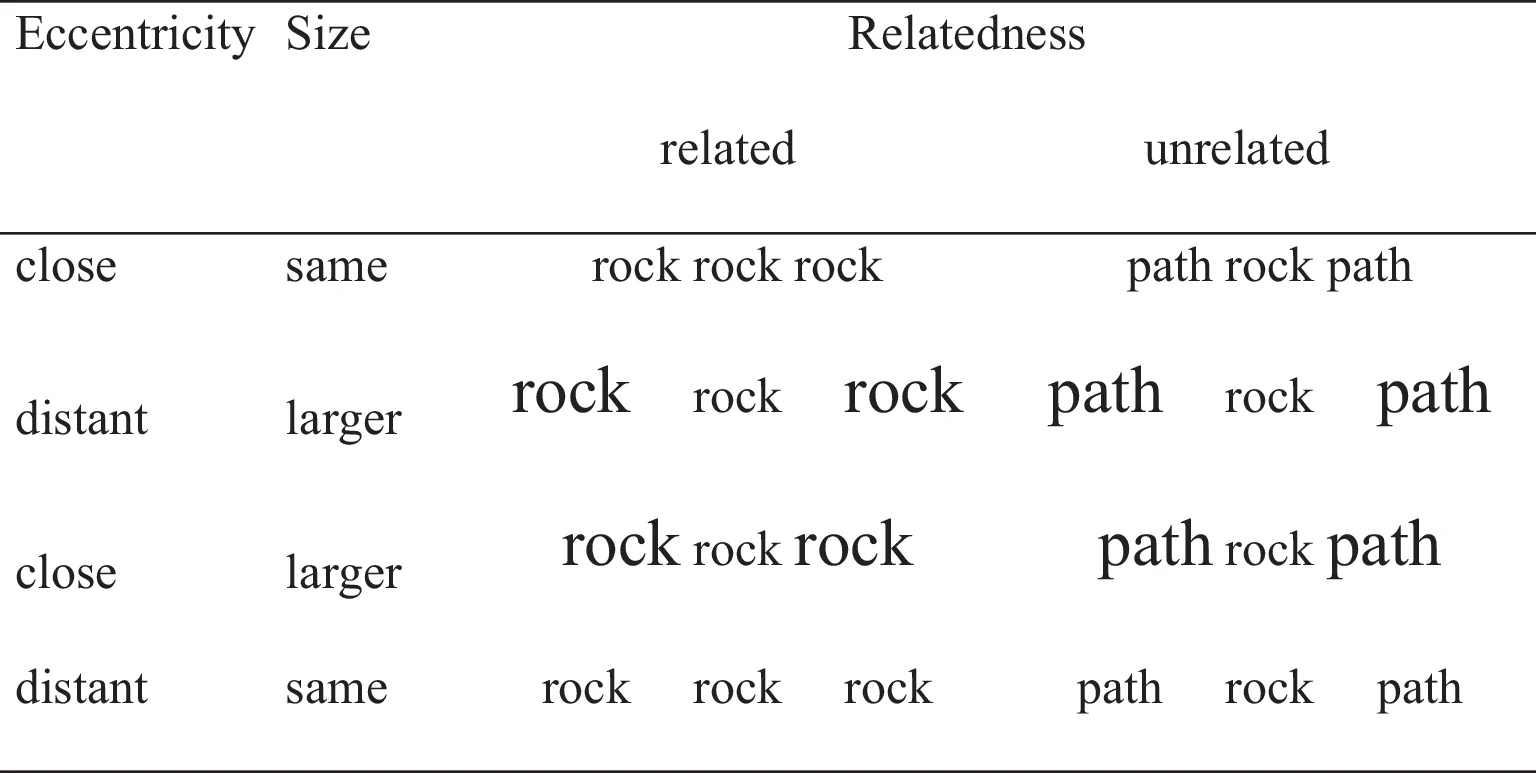
Examples of target and flanker stimuli in the eight experimental conditions tested in the present study

Larger eccentricity (distant condition) is depicted here as five character spaces rather than the use of seven spaces in the actual experiment. Note also that the change in letter size in the actual experiment was from 16 to 24 points

Following the results of Fournet et al. (2023) and Cauchi and Meeter (2025), we expected to observe an interaction between relatedness and eccentricity, with smaller effects of flanker relatedness when flankers are more distant from targets. However, the main aim of the current study was to determine whether an increase in flanker size could compensate for the effects of flanker eccentricity. That is, could increasing the size of the more distant flankers help increase their impact on target word processing. Such a result would provide a further demonstration of the role played by purely visual factors in the reading version of the flankers task. Moreover, we provide a comparison of two visual factors – eccentricity and size – and we examine possible interactions between these factors, as well as their interactions with flanker relatedness.

## Methods

### Participants

One hundred and sixty native French speakers (66 women) participated in a 30-min online experiment using their personal computers. The ages of the participants ranged from 19 to 75 years (*M* = 38.80 years, *SD* = 12.7). In this experiment, participants received £10 per hour as compensation (via the Prolific platform). The purpose of the experiment was not disclosed to the participants. Prior to the start of the experiment, participants were informed that the data would be collected anonymously, and then gave informed consent to participate, as well as information about their age, first language, and gender. Finally, seven participants were excluded because their performance was more than 2.5 *SD*s beyond the grand mean.^1^

### Stimuli and design

We selected a set of 160 target-flanker pairs of French words that served for the unrelated conditions (in the related conditions, targets and flankers were the same word). All these words were selected from the Manulex lexical database (Lété et al., 2004). They were four letters long and did not contain letter repetitions given that letter repetitions are known to affect word recognition (Kerr et al., 2021; Trifonova & Adelman, 2019). Furthermore, since the role of diacritics in reading is still poorly understood, we decided not to include words with diacritics in this study. To reduce the impact of foveal load effects (e.g., White et al., 2005), targets were systematically more frequent than flankers. In the initial 160 target-flanker word pairs, 80 pairs had a high lexical frequency (target mean zipf = 3.71, *SD* = 0.52; flanker mean zipf = 3.67, *SD* = 0.50) and 80 pairs had a low lexical frequency (target mean zipf = 2.33, *SD* = 0.46; flanker mean zipf = 2.31, *SD* = 0.47). The frequency factor served for the word selection but was not retained as a factor in the final analysis. The creation of target-flanker pairs avoided as much as possible sharing letters between the target and flanker. From this set of 160 items, we constructed eight experimental conditions, as presented in Table 1. The pseudoword set was created from the word set by single-letter substitution. As with the word set, we avoided having target and flanker share letters as much as possible. The OLD20 (Yarkoni et al., 2008) values of these pseudowords were 1.79 (range = 1–2.85), which is a good value for optimizing participants’ response times (RTs) on the word set.

A 2 × 2 × 2 design was retained with Relatedness (related vs. unrelated), Size (same vs. larger), and Eccentricity (close vs. distant) as within-participant factors. Eight experimental lists were created so that the 160 words and 160 pseudowords could be tested under all experimental conditions. There were 20 words per condition per participant.

### Apparatus

The experiment was created with Labvanced (Finger et al., 2017) and we used the Prolific platform (Pallan & Schitter, 2018) to recruit participants.

### Procedure

**Fig. 1:**
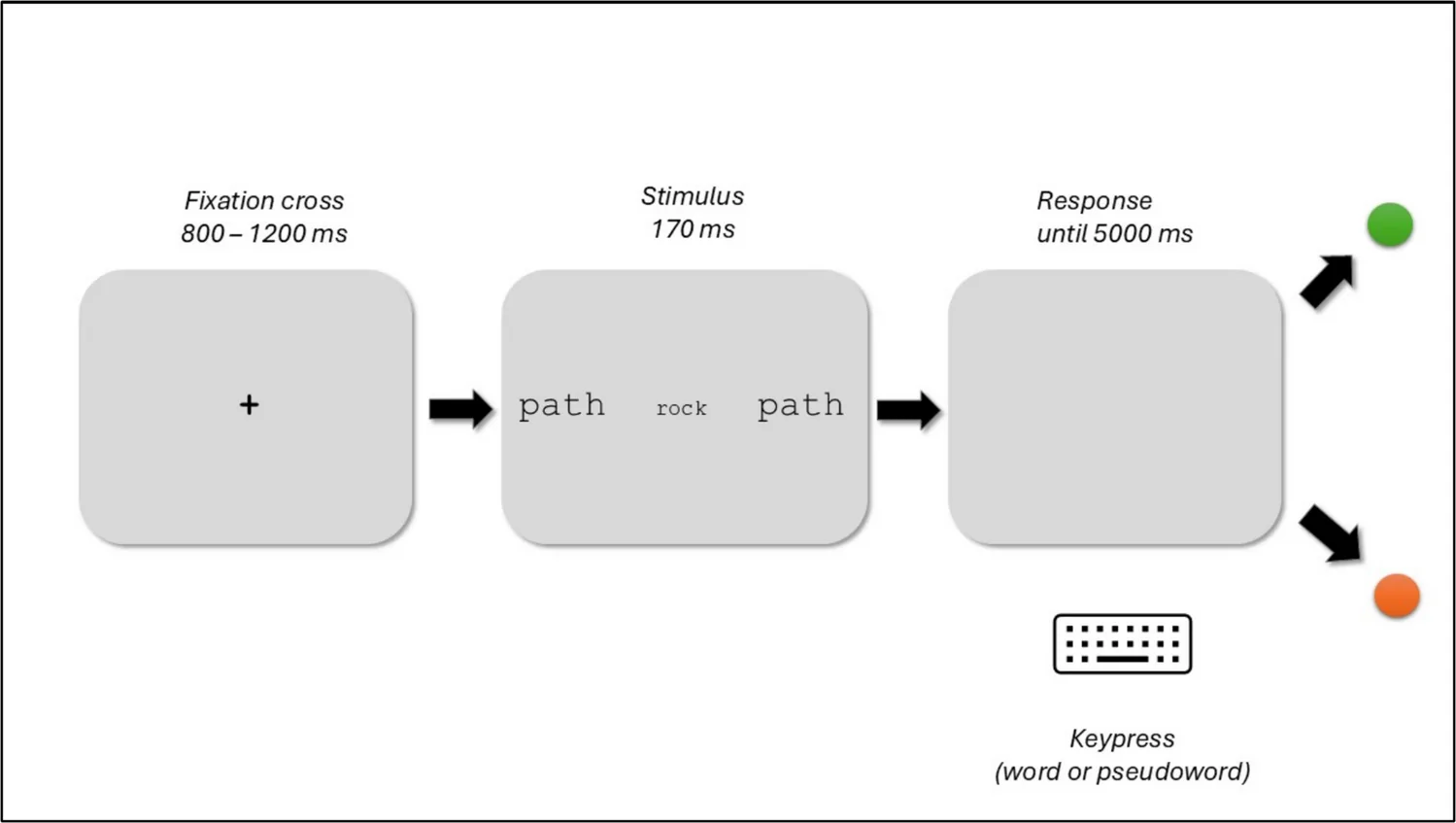
Experimental procedure of one trial with an example of the unrelated-distant-larger flanker condition. The size and distances used in this figure are only for illustration (see Methods section for precise information), and the example stimuli are given in English for convenience

Participants were asked to click on the screen to start the experiment. They were then shown the full instructions for the experiment. After reading and understanding the instructions, the participant could start the practice trials by pressing the space bar. The practice session consisted of 16 trials that were representative of the eight conditions tested in the experimental session but were not included in this session. Upon completion of the practice session, participants were asked to press the space bar when they were ready to begin the experimental session. Participants were instructed to determine as quickly and accurately as possible whether or not the four-letter string in the center was a correct French word. Each trial started with a fixation cross for a variable time between 800 and 1,200 ms, indicating the center of the upcoming sequence (target plus flankers), and participants were instructed to fixate this fixation cross. After the fixation cross disappeared, the string of letters was presented for 170 ms, followed by a blank screen displayed for a maximum of 5,000 ms to await the participant’s response. As is typical for experiments using the flanker task, a short stimulus presentation time (target + flankers) was used to minimize the possibility of eye movements away from the central fixation cross and towards the flankers. Participants were instructed to press the right arrow key on their computer keyboard if they thought the central string of letters was a correct French word, or the left arrow if it was not. Feedback was provided in the form of a green dot for a correct response and a red dot for an incorrect response (Fig. 1).

### Statistical power

The remaining sample size of 153 participants and 153 items substantially exceeded Brysbaert and Stevens’ (2018) recommendations of a minimum of 1,600 datapoints per condition for within-subject designs with crossed subjects and items. The sample size (*n* = 153 participants; *n* = 20 items per condition) yielded 3,060 data points per cell before data exclusions. To ensure that the study was adequately powered, we used the simulation approach proposed by the same authors. In this way, we used the Monte Carlo method with the powerSim function from the simR package (version 1.0.5; Green & MacLeod, 2016). At each iteration, the algorithm selected a random sample of 117 participants and 117 items, representing a 25% reduction from the original dataset, and fitted a linear mixed effects model. The significance of the Relatedness by Eccentricity interaction was then tested across 40 simulations per iteration, for a total of 200 iterations. This procedure aimed to capture the variability in power estimates due to sample selection and resulted in an estimated statistical power of 100%. Given the standard of 80% for adequate power, this analysis suggests that the study was adequately powered.

## Results

Data concerning the pseudoword fillers were not analyzed. Error trials (5.6%) and trials with unusually short or long RTs (below 300 ms or above 3,000 ms: 1.1%) were excluded prior to analysis. RTs were log-transformed to meet normality assumptions. This transformation was applied to improve the approximation of normality of model residuals, as assumed by linear mixed-effects models.^2^ A null model with a random structure including random intercepts by participant and by item was fitted to compute standardized residuals from the log RTs variable. All trials with standardized residuals larger than 2.5 *SD*s from the grand mean were excluded (2.5%). To avoid a speed-accuracy trade-off caused by rare words, all items with an accuracy rate or RT mean larger than 2.5 *SD*s were discarded. This affected one item in the frequent words set and six items in the rare words set. The remaining set consisted of 153 items.^3^

**Fig. 2:**
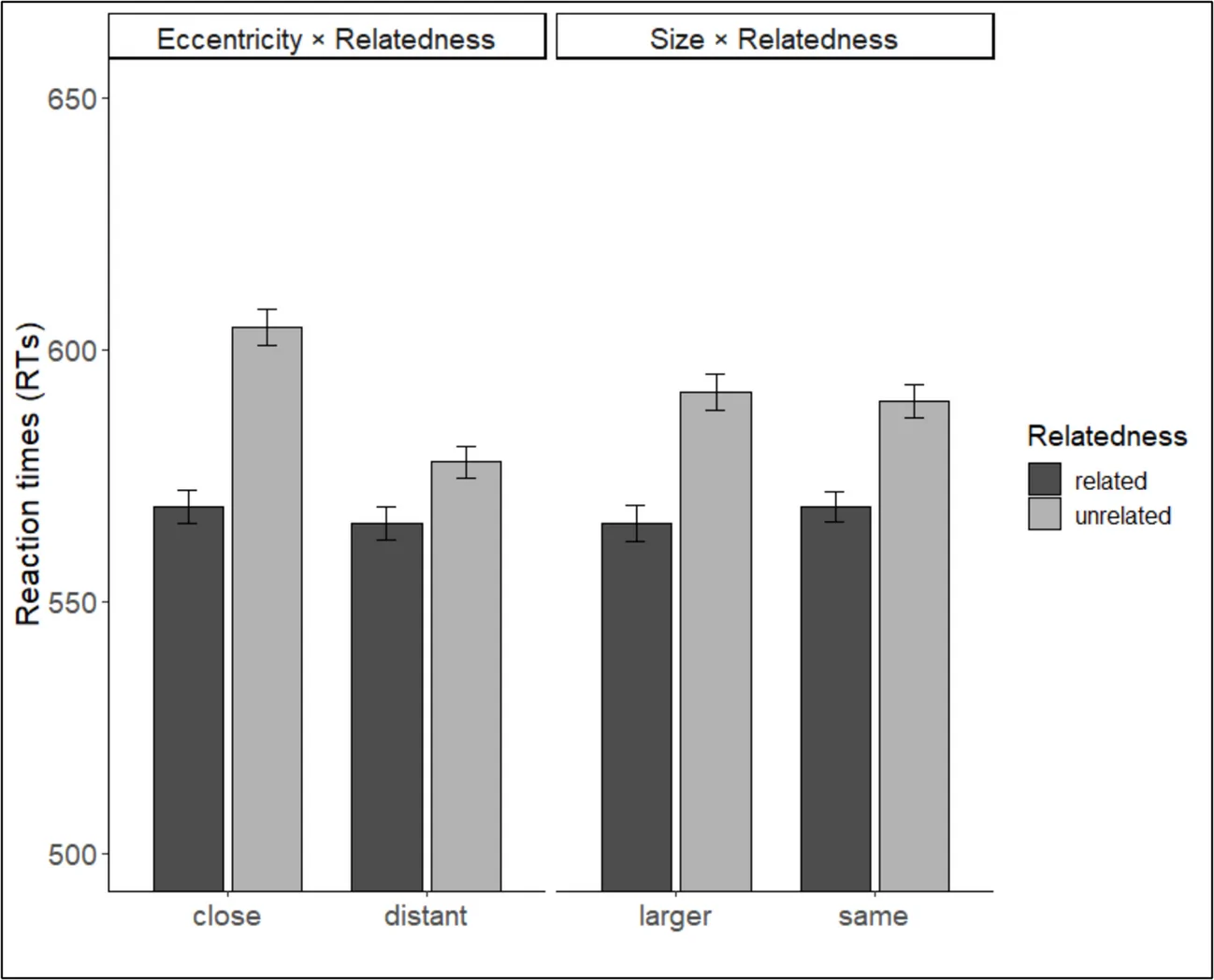
Mean response times (RTs, in ms) illustrating the significant two-way interactions: flanker relatedness × flanker eccentricity (left panel) and flanker relatedness × flanker size (right panel). Error bars are within-participant 95% confidence intervals (Cousineau & O’Brien, 2014)

The data were analyzed in the R statistical computing environment (R Core Team, 2022). For the RT analyses, linear mixed-effects models were fitted using the *lmer* function from the lme4 package (version 1.1–21; Bates et al., 2014). For the accuracy rate analyses, where accuracy is a binomial variable, generalized mixed effects models were fitted using the *glmer* function from the above package. For both RT and accuracy rate analyses, the random effects structures included items and participants as crossed random effects. For each model, the maximum random effects structure that converged was retained to justify the fixed effect structure while avoiding overfitting (Barr et al., 2013). Regression coefficients (b), standard errors (*SE*s) and t-values (for the linear mixed effects model) and z-values (for the generalized mixed-effects model) are presented below. Fixed effects were considered significant if |t| or |z|> 1.96 (Baayen, 2008). The following section presents firstly the analyses of RTs and secondly the analyses of accuracy rates. Only significant terms are reported. The full fixed effects from the mixed-effects models are reported in the Appendix. Mean RTs per condition are plotted in Fig. 2.

### Response times

For the three-way interaction model, the maximal converging random effect structure included by-participant and by-item random intercepts and slopes for the Flanker Relatedness, Eccentricity, and Size factors. In this analysis, the main effect of Relatedness was significant (*b* = −9.32, *SE* =.68, *t* =—13.77), indicating faster RTs in the related flanker conditions (567 ms) compared to the unrelated conditions (591 ms). The main effect of Eccentricity was also significant (*b* = 5.96, *SE* =.53, *t* = 11.21), with faster RTs observed with distant flanker conditions (571 ms) compared to the close flanker condition (585 ms). The Relatedness by Eccentricity interaction was significant (*b* = −4.64, *SE* =.47, *t* = −9.91), with a relatedness effect of 35 ms in the close flanker condition and a 12-ms effect in the distant flanker condition. The Relatedness by Size interaction was also significant (*b* = −1.14, *SE* =.47, *t* = −2.43), with a relatedness effect of 27 ms in the same-size flankers condition and a 21-ms effect in the larger-size flankers condition. A complementary Bayesian model comparison between a full model including the Relatedness by Size interaction and a null model without this interaction yielded a Bayes factor of BF_10_ =.39 (BF_01_ = 2.55), indicating weak evidence in favor of H0. The Relatedness by Eccentricity by Size interaction was not significant (*b* = 3.81, *SE* =.47, *t* = 0.81). The full pattern of effects is presented in the Appendix.

### Accuracy

**Fig. 3:**
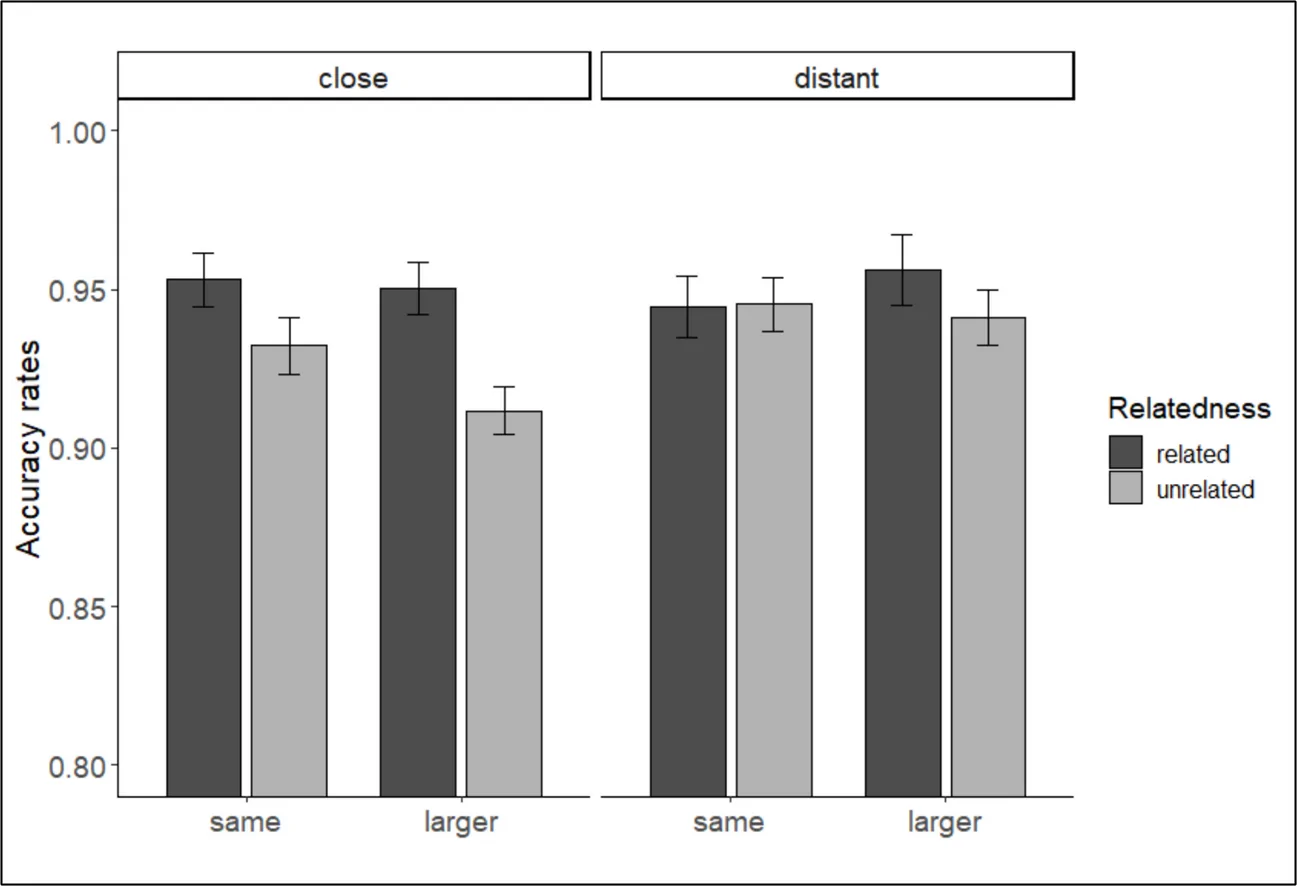
Mean accuracy (%) per experimental condition: flanker relatedness (related vs. unrelated) × flanker eccentricity (close vs. distant) × flanker size (same vs. larger). Error bars are within-participant 95% confidence intervals (Cousineau & O’Brien, 2014)

Mean accuracy per experimental condition is shown in Fig. 3. For the three-way interaction model, the maximal converging random effect structure was one including by-participant and by-item random intercepts and slopes among the Relatedness Factor. In the analysis of accuracy rates, the main effect of Relatedness was significant (*b* = -.67, *SE* =.12, *z* = −5.60), with an accuracy of 95% in the related flanker condition compared to 93% in the unrelated flanker condition. The Relatedness by Eccentricity interaction was significant (*b* =.38, *SE* =.17, *z* = 2.23), with a relatedness effect of 2.9% in the close flanker condition and an effect of 0.7% in the distant flanker condition. The Eccentricity by Size interaction barely reached significance (b = -.37, SE =.18, z = −2.03), with an effect of 1.1% in the close flanker condition and an effect of 0.4% in the distant flanker condition. The Relatedness by Size interaction was not significant. The three-way interaction was not significant. The full pattern of effects is presented in the Appendix.

## Discussion

In the present study we set out to examine if an increase in flanker size could compensate for the reduced effects of flanker relatedness when flankers are located further from targets. We knew from prior studies that an increase in flanker eccentricity causes a significant reduction in the size of effects of flanker relatedness (Cauchi & Meeter, 2025; Fournet et al., 2023). This effect of flanker eccentricity occurs as long as other flankers do not intervene between the related flanker and target. That is, the evidence so far suggests that only adjacent flankers impact on performance to central target words (Fournet et al., 2023; Mlinarič et al., 2025). We therefore did not introduce intervening flankers in the present study, and flanker eccentricity was manipulated by increasing the spacing between targets and flankers.

Replicating prior research (Cauchi & Meeter, 2025; Fournet et al., 2023), we found a significant interaction between flanker eccentricity and flanker relatedness, with the relatedness effect being greatly reduced with the distant flankers (see Figs. 2 and 3). One novel finding of the present study is that increasing the size of flankers was found to increase effects of flanker relatedness, with larger flankers producing significantly greater effects of flanker relatedness, although this increase in effects of flanker relatedness was quite small (6 ms in RTs and 1% in accuracy rates). This is likely due to the greater visibility of the larger flanking stimuli, with this greater visibility providing a stronger input to the process of spatial integration of orthographic information (i.e., stronger activation of orthographic information derived from flankers – hence the stronger effects of flanker relatedness). Inspection of Figs. 1 and 2 suggests that the observed interactions are primarily driven by increased interference in the unrelated flanker conditions rather than by facilitation in the related conditions. RTs and accuracy in the related conditions were relatively stable across the size and eccentricity manipulations, while the opposite was true for the unrelated conditions. This pattern is consistent with the idea that orthographic flanker effects reflect the combination of a general interfering effect of the presence of orthographic flankers, and a reduction in this interference in the related flanker condition. Massol et al. (2026) provided support for this interpretation in a study that included a non-letter flanker condition (ampersand flankers). Performance in this non-letter flanker condition did not significantly differ from the related bigram flanker condition and was significantly better than the unrelated bigram flanker condition.

The key finding of the present study is that increasing flanker size did not compensate for a decrease in effects of flanker relatedness with increasing flanker eccentricity. That is, the three-way interaction between flanker relatedness, flanker size, and flanker eccentricity was not significant, either in RTs or in error rates. This demonstrates that the impact of purely visual factors on effects of flanker relatedness is not cumulative (i.e., increasing flanker size does not modify the effects of flanker eccentricity on effects of flanker relatedness), and that other factors, most notably attentional factors (e.g., Fournet et al., 2023; Lázaro et al., 2026; Snell & Grainger, 2018), could be at play in determining the magnitude of flanker relatedness effects.

The present findings align nicely with the results of studies manipulating letter size during single word recognition (Nazir et al., 1998). Individual letter size was manipulated with the aim to compensate for a drop in letter visibility as a function of eccentricity from fixation location in the word. As in the present study, this manipulation did not work – that is, increasing the size of more eccentric letters did not compensate for their reduced impact on word recognition (Nazir et al., 1998). Following Nazir et al.’s (1998) interpretation of their results, one factor that might cancel the benefits of larger flanker size with greater flanker eccentricity is that everyday reading does not involve changes in the font size of words, in the same way that letter size does not change within a word during everyday reading. Nazir et al. appealed to reading habits as the underlying cause of the failure to improve word recognition by increasing the size of letters the further they are from fixation. This generates a format that is atypical and thus interferes with word recognition. The same reasoning could be applied to the present results, with the atypical change in size between targets and flankers interfering in the spatial integration of information across targets and flankers.

However, our preferred interpretation of the present findings is that the increase in flanker size results in a greater attraction of spatial attention to flanker stimuli at the expense of attentional resources allocated to target processing (i.e., a wider spread in spatial attention with larger flankers). We hypothesize that this distribution of attentional resources across targets and flankers would have the greatest impact with distant flankers and would have a lesser impact with close flankers. We therefore predict a graded increase in the impact of flanker size as flanker eccentricity is increased incrementally. This is a prediction to be tested in future research.

## Supplementary Information

Below is the link to the electronic supplementary material.

Supplementary file1 (DOCX 29 kb)

## Data Availability

The data, materials, and code for statistical analyses are available through Open Science Framework at https://osf.io/ebma8/

## Footnotes

1 Five participants were removed due to high error rates (> 20%; > 2.5 *SD*s from the sample mean), and two participants were removed due to slow RTs (> 860 ms; > 2.5 *SD*s).

2 The robustness of the results was verified using alternative modelling approaches. First, generalized linear mixed-effects models with a Gamma distribution were fitted to the RT data, yielding the same pattern of significant effects as the log-transformed models. Second, linear mixed-effects models on untransformed RTs using the same fixed- and random-effects structure were fitted. Again, in this second analysis, the overall pattern of effects remained unchanged.

3 In order to provide a minimal analysis, the analysis was repeated using progressively stricter criteria: minimal trimming(exclusion of extreme RTs), residual screening, item screening, and participant screening (final model). Across all four levels ofpre-processing, the main effects of Relatedness and Eccentricity, and the Relatedness × Eccentricity interaction, remainedsignificant. The Relatedness × Size interaction was not significant in either the minimal trimming or the residual screening.However, this interaction reached significance in both the item screening model and in the participant screening model, asreported in the Results.

## Code availability

Code and data are available via the Open Science Framework at https://osf.io/ebma8/

## Declarations

### Open practices statement

We have provided trial-level data for the experiment, along with experiment materials (words with flankers) and statistical analysis code. None of the experiments were preregistered.
