# Supplementary material for: Effects of flanker size and flanker eccentricity on the spatial integration of orthographic information
Source: Atten Percept Psychophys. 2026 Jun 11;88(5):147. doi: 10.3758/s13414-026-03293-w (PMC13260275; doi:10.3758/s13414-026-03293-w)
Supplement: Supplementary file 1 — Supplementary file1 (DOCX 29 kb) [file 13414_2026_3293_MOESM1_ESM.docx]

**Appendix**

Complete results of the mixed-effects models for response times (Table A1) and accuracy (Table A2)

**Table A1** Results of the linear mixed-effects model for response times (RTs)

| **Fixed effects** | **Estimate** | **Std. error** | **df** | ***t* value** | ***p* value** |
| --- | --- | --- | --- | --- | --- |
| Intercept | 2753 | 5.08 | 189.4 | 542.35 | < .001 |
| relatedness | -9.32 | 0.68 | 135.5 | -13.77 | < .001 |
| eccentricity | 5.96 | 0.53 | 122.5 | 11.21 | < .001 |
| Size | -0.05 | 0.50 | 142.0 | -0.09 | >.10 |
| Relatedness × eccentricity | -4.64 | 0.47 | 20630 | -9.91 | < .001 |
| Relatedness × size | -1.14 | 0.47 | 20590 | -2.43 | <.05 |
| Eccentricity × size | 0.81 | 0.47 | 20580 | 1.73 | .083 |
| Relatedness × eccentricity × size | 0.38 | 0.47 | 20580 | 0.81 | >.10 |

*Note.* Estimates and standard errors are reported multiplied by 1,000 for readability

**Table A2** Results of the generalized mixed-effects model for accuracy

| **Fixed effects** | **Estimate** | **Std. error** | ***z* value** | ***p* value** |
| --- | --- | --- | --- | --- |
| Intercept | 3.54 | 0.13 | 26.90 | < .001 |
| Relatedness | -0.67 | 0.12 | -5.60 | < .001 |
| Eccentricity | 0.11 | 0.13 | 0.78 | >.10 |
| Size | 0.08 | 0.13 | 0.59 | >.10 |
| Relatedness × eccentricity | 0.38 | 0.17 | 2.23 | <.05 |
| Relatedness × size | 0.17 | 0.17 | 1.04 | >.10 |
| Eccentricity × size | -0.37 | 0.18 | -2.03 | <.05 |
| Relatedness × eccentricity × size | 0.13 | 0.24 | 0.54 | >.10 |
